# Are there gender-specific differences in hip and knee cartilage composition and degeneration? A systematic literature review

**DOI:** 10.1007/s00590-024-03871-4

**Published:** 2024-03-08

**Authors:** Alberto Di Martino, Francesca Barile, Claudio D’Agostino, Vanita Castafaro, Tosca Cerasoli, Paolo Mora, Alberto Ruffilli, Francesco Traina, Cesare Faldini

**Affiliations:** 1 Department of Biomedical and Neuromotor Sciences (DIBINEM), Alma Mater Studiorum University of Bologna, Bologna, Italy; 2 Ist Orthopedic Department, IRCCS – Istituto Ortopedico Rizzoli, Via G. Cesare Pupilli, 1, Bologna, Italy; 3 Ortopedia-Traumatologia e Chirurgia Protesica e dei Reimpianti d’Anca e di Ginocchio, IRCCS – Istituto Ortopedico Rizzoli, Bologna, Italy

**Keywords:** Sex difference, Gender medicine, Osteoarthritis, Cartilage volume, Cartilage morphology, Cartilage composition

## Abstract

The aim of the present review is to systematically analyse the current literature about gender differences in hip or knee cartilage composition and degeneration, to help explaining how and why osteoarthritis affects women more often and more severely than men. A systematic review of the literature in English was performed. Eleven studies on 1962 patients (905 females and 787 males) that reported differences on cartilage composition between males and females were included. Nine evaluated the knee, one the hip, and one both. They were heterogeneous in their methods: one conducted histological analyses, and all the others evaluated cartilage characteristics (volume, width, and composition) through magnetic resonance imaging. All authors reported gender differences in both volume and morphology of the cartilage, from infancy to menopause. In fact, a study on 92 healthy children statistically showed significant gender differences in cartilage thickness at all sites, even after adjustment for age, body, and bone size. Gender differences become more evident after menopause, when women have a lower cartilage volume and a higher cartilage loss. Men show significantly higher knee and hip cartilage volumes than women, and women carry a significantly greater risk to develop osteoarthritis. This is in part due to body and bone size, but also depends on qualitative and quantitative differences in the composition of cartilage and its degeneration rate after menopause. Structural changes in cartilage that occur between genders during ageing have significance in the development of osteoarthritis.

## Introduction

The most common musculoskeletal illness, osteoarthritis (OA), affects more than 300 million individuals globally and is a primary cause of disability [1]: 10–15% of all adults over the age of 60 have some type of OA [2]. Despite the scientific community's efforts, the processes underlying the genesis, evolution, and therapy of OA remain unknown. While risk variables such age, genetic profiles, obesity, and traumatic injury [3] have been discovered and established, the role of gender has been undervalued [4, 5]. As a matter of facts, there are sex/gender variations in the frequency, incidence, and severity of OA: women are more affected, particularly following menopause, and often exhibit more severe clinical symptoms [6]. A meta-analysis of nine studies that provide impact sizes for gender on risk variables for the start of knee OA found that females have an odds ratio of 1.84 (95% CI 1.32–2.55) compared to males [7]. An explanation for this difference may lie in the distinct cartilage compositions between men and women, which may predispose female to a sooner joint degradation [8, 9]. Imaging methods like magnetic resonance imaging (MRI) allow for non-invasive evaluation of cartilage composition, loss of collagen matrix and proteoglycans and water content variation [10, 11].

The purpose of this study is to conduct a systematic review of the available studies on gender differences in hip or knee cartilage composition and degeneration. In fact, a study that lays the foundation for the different clinical manifestations of OA in men and women is lacking to date. This study could help to understand the structural phenomena (quantitative and qualitative change of the cartilage) underlying the different clinical manifestations of OA between men and women. Knowing the changes in cartilage structure could also be helpful in laying the foundation in the prevention of joint arthrosis and developing successful tailored diagnostic and treatment solutions.

## Materials and methods

### Search strategy

**Fig. 1:**
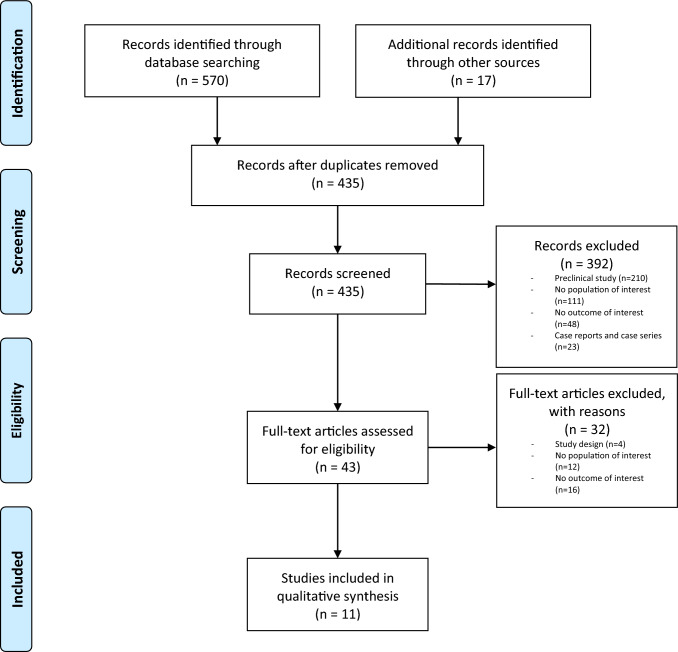
PRISMA algorithm: search strategy according to the Preferred Reporting Items for Systematic Reviews and Meta-Analyses (PRISMA) statement

In April 2023, a comprehensive evaluation of the existing English literature was conducted using three big databases (Scopus, Embase, and PubMed). The strings MeSH terms utilized were a combination of the following keywords: “hip”, “knee”, “cartilage characteristics”, “gender”, “sex”, “cartilage composition”, “differences”, “osteoarthritis”. Two authors independently screened all potentially relevant titles and abstracts, and any disagreement was solved by the senior authors. During the development of this review, the Preferred Reporting Items for Systematic Reviews and Meta-Analyses guidelines were followed [12] (Fig. 1).

Two authors independently assessed the quality of included trials using the NIH tool [13]. Two of the authors retrieved reference data, populations, and outcomes from the papers into pre-specified tables with Excel (Microsoft^®^). The authors extracted data on the basic features of the studies (including the design and primary outcomes), participants (population and sample size), treatments (diagnostic modality of cartilage composition), comparator (if applicable), and summary of major results. An attempt was made to compare qualitative and quantitative differences in articular cartilage of the knee and of the hip between male and female with the methods used in individual studies.

### Eligibility criteria

For the current study, the PICOS model (Population, Intervention, Comparison, Outcomes, Study design) was used: studies that considered healthy subjects and/or patients with hip or knee osteoarthritis (Population), submitted or did not submit to a specific surgical intervention (Intervention), with or without a comparison group of healthy controls (Comparison), reported differences in cartilage composition between males and females (Outcomes), in clinical studies (Study design).

There were no publishing year restrictions. Only articles in English were considered. The search was limited to human data. Reviews, case reports, case series, and in vitro or in vivo studies were eliminated; randomized controlled trials (RCTs), retrospective or prospective observational studies that satisfied the indicated PICOS were included.

## Results

### Baseline characteristics

The search for relevant publications retrieved 587 studies. These references were entered into a reference manager (Mendeley 1.14) and duplicates (*n* = 152) were eliminated. The remaining 435 records were examined for title, abstract, and full text. In the end, 11 papers were selected for synthesis (Fig. 1). The key aspects of these investigation are extracted and summarized in Table 1. There were eight cross-sectional studies (CSS) [14–21], two prospective studies (PS) [22, 23], and one longitudinal cohort study (LCS) [24] among the 11 publications chosen. They studied 1692 patients, 905 females and 787 males. According to the NIH tool, all the studies were of high quality (Table 2).

**Table 1:** Studies characteristics

References	Design and population (M/F)	Aim	Methods	Cartilage evaluation	Main results	Major findings
Kaspiris et al. [14]	CSS 20 (15/5)	To examine frequency of cartilage’s cracks and correlate them with the cellular modifications	Histological analysis was conducted on nine specimens per patient	Histological analysis	No association between the number of cracks and age or BMI	Combination of hormonal factors and less bone density in women may be responsible for cartilage microcracks
Berman et al. [20]	CSS 220 (110/110)	To consider relative defect size as opposed to absolute defect size when treating osteochondral lesions of the knee	After obtaining 3 T MRI for all patients, the femoral condylar articular width was measured from reproducible anatomic locations	MRI	There is a statistically significant difference between males and females’ cartilage width	Male showed a bigger cartilage width compared to female
Nemeth et al. [22]	PS 30 (16/14)	To assess the T1p and T2 values in the hip cartilage of healthy volunteers	Quantitative 3D T1q- and T2-maps sequences of the right hip. The same was repeated 14 days later	MRI	The mean of T1q values was 6.0 ms higher in women than in men	There is a higher risk of cartilage degeneration in women
Antony et al. [21]	PS 190 (99/91)	To describe the association between body composition and hormonal factors	Tibial cartilage volume, SHBG, testosterone and CRP level and fibrinogen in both sexes were measured 5 years prior	MRI	Men had 13% more tibial cartilage volume (500 mm^3^) than women	Sex difference in knee cartilage volume is contributed largely by variations in body composition and/or fibrinogen
Kumar D et al. [16]	CSS 184 (81/103)	Evaluate knee articular and meniscus cartilage T1q relaxation times	T1q and T2 relaxation times for cartilage in the knee were quantified with 3 T MRI	MRI	Women had higher lateral articular cartilage T1q and patellofemoral T1q in the OA group; and higher lateral meniscus T1q in the young group	Women have worse cartilage composition with fewer proteoglycans
Pachowsky et al. [23]	PCS 40 (20/20)	To determine morphological and biochemical gender related differences in cartilage repair tissue	Patients were examined clinically and by MR scans at 3 T-MRI for assessment of healthy cartilage and MACT cartilage	MRI and clinical evaluation	There is a different T2 values between cartilage of male and female	Gender differences in cartilage quality are seen, showing a less ability of the female cartilage to repair
Tameem et al. [15]	CSS 60 (30/30)	To create an average atlas of knee femoral cartilage morphology	An atlas was created based on images from 30 male Caucasian subjects with no symptomatic OA at baseline	MRI	After global scaling to the male template, the female cartilage resulted to be thicker in most regions	If the cartilage scales nonlinearly with body size, then a linear global correction would result in a bigger cartilage in women
Ding et al. [24]	LCS 325 (135/190)	To describe the association between sex, age and rate of cartilage volume change in knee	Cartilage volume and bone size were determined using MRI. Height, weight, BMI, and radiographic OA were measured	MRI	Females had higher rates of knee cartilage volume loss per year, more marked after age 40	Women have substantially higher knee cartilage loss than men
Ding et al. [19]	CSS 372 (158/213)	Sex differences in knee cartilage volume may be mediated through body and bone size, age and/or physical activity	Articular cartilage volumes and bone size were determined by MRI. Height, weight, physical activity, and radiographic OA were measured	MRI	Gender explained 33–42% of the variation in knee cartilage volumes volume at all sites	Men have higher knee cartilage volumes than women
Faber et al. [18]	CSS 18 (9/9)	To compare cartilage thickness, volume, and articular surface areas of the knee in male and female	Cartilage thickness quantification in healthy subjects	MRI	Females showed smaller cartilage volumes than males	Differences in cartilage volume are primarily due to differences in joint surface areas
Jones et al. [17]	CSS 92 (49/43)	To evaluate if sex-related differences in cartilage development might be related to the risk of knee OA	Articular cartilage thickness and volume were determined at the patella, medial and lateral tibial compartments	MRI	Sex accounted for 6–36% of the variation in cartilage thickness and volume Males more than females	Cartilage sex-related differences may be one explanation for knee OA seen in later life

*CSS* cross-sectional study, *PS* prospective study, *LCS* longitudinal cohort study, *OA* osteoarthritis, *MRI* magnetic resonance imaging, *BMI* body mass index, *MACT* matrix-associated autologous chondrocyte transplantation, *SHBG* sex hormone binding globulin, *CRP* c reactive protein

**Table 2:** Quality of studies

References	Was the study question or objective clearly stated?	Was the study population clearly and fully described, including a case definition?	Were the cases consecutive?	Were the subjects comparable?	Was the intervention clearly described?	Were the outcome measures clearly defined, valid, reliable, and implemented consistently across all study participants?	Was the length of follow-up adequate?	Were the statistical methods well-described?	Were the results well-described?	Quality summary
Kaspiris et al. [14]	✔	✔	✔	✔	✔	✔	✗	✔	✔	2
Berman et al. [20]	✔	✔	✔	✔	✔	✔	✗	✗	✔	2
Nemeth 2017	✔	✔	✔	✔	✔	✔	✗	✔	✔	2
Antony et al. [21]	✔	✔	✔	✔	✔	✔	✔	✔	✔	2
Kumar et al. [16]	✔	✔	✔	✔	✔	✔	✗	✔	✗	2
Pachowsky et al. [23]	✔	✔	✔	✔	✔	✔	✔	✗	✔	2
Tameem et al. [15]	✔	✔	✔	✔	✔	✔	✗	✔	✔	2
Ding et al. [24]	✔	✔	✔	✔	✔	✗	✔	✗	✔	2
Ding et al. [19]	✔	✔	✔	✔	✔	✔	✗	✔	✔	2
Faber et al. [18]	✔	✔	✔	✔	✔	✔	✗	✔	✔	2
Jones et al. [17]	✔	✔	✔	✔	✔	✔	✗	✔	✔	2

Quality was rated as 0 for poor (0–3 out of 9 questions), 1 for fair (4–6 out of 9 questions), or 2 for good (7–9 out of 9 questions)

*NA* not applicable, *NR* not reported

Different procedures were used in the studies examined. In terms of cartilage evaluation, only one author performed histological studies [14]. The other studies [15–24] assessed its composition (volume, length, width, T1/T2 values) through MRI. Nine studies looked at the knee [15–21, 23, 24], one at the hip [22], and one at both joints [14]. Table 3 presents a summary of the main results obtained from the various studies.

**Table 3:** Summary of the main findings of the papers analysed

References	Study	Most important results
Jones et al. [17]	Sex and site differences in cartilage development: A possible explanation for variations in knee osteoarthritis in later life	Gender explained 6–36% of the difference in cartilage thickness and volume of the knee
Faber et al. [18]	Gender differences in knee joint cartilage thickness, volume and articular surface areas: Assessment with quantitative three-dimensional MR imaging	Women had 19.9% less patellar cartilage and 46.6% less medial tibial cartilage than males
Ding et al. [24]	Sex differences in knee cartilage volume in adults: Role of body and bone size, age and physical activity	Men have considerably greater knee cartilage volume (33–42%) than women
Ding et al. [19]	A longitudinal study of the effect of sex and age on rate of change in knee cartilage volume in adults	After 40 years of age, women had a much higher incidence of cartilage deterioration than men (medial tibia -3.5%, lateral tibia –2.6% and patella -0.8% of cartilage loss per year)
Tameem et al. [15]	Initial results on development and application of statistical atlas of femoral cartilage in osteoarthritis to determine sex differences in structure: Data from the Osteoarthritis Initiative	Women had thicker cartilage (average Jacobian, a voxel-based measure of volume that gives information on localized volume changes) at 1.2 ± 0.078 vs. 1.08 ± 0.097
Pachowsky et al. [23]	3D-isotropic high-resolution morphological imaging and quantitative T2 mapping as biomarkers for gender related differences after matrix-associated autologous chondrocyte transplantation (MACT)	T2 values of joint cartilage using MRI were substantially shorter in males than in women
Kumar et al. [16]	Are there sex differences in knee cartilage composition and walking mechanics in healthy and osteoarthritis populations?	Women had larger amounts of lateral articular and patellofemoral cartilage on T1ρ, indicating lesser proteoglycan content and tissue quality
Berman et al. [20]	Gender disparity between absolute versus relative size of condylar chondral defects: An MRI analysis	There is a statistically significant difference in articular cartilage width between males and females (31.62 ± 3.54 for males and 26.53 ± 3.70 for females)
Anthony et al. [21]	Association of body composition and hormonal and inflammatory factors with tibial cartilage volume and sex difference in cartilage volume in young adults	Men's tibial cartilage volume was determined to be 13% larger than women's by MRI
Nemeth et al. [22]	Reproducibility of in vivo magnetic resonance imaging T1 rho and T2 relaxation time measurements of hip cartilage at 3.0 T in healthy volunteers	Females had a higher average T1ρ value than males (5.98 ms)
Kaspiris et al. [14]	Sex, but not age and bone mass index positively impact on the development of osteochondral micro-defects and the accompanying cellular alterations during osteoarthritis progression	Histologically, cartilage of women presents more cracks compared to men, showing a worse quality

Higher quality and quantity of articular cartilage in men than in women is confirmed in all studies, except in Tameem

### Knee cartilage morphometry

Most of the included research discussed how gender differed in terms of knee morphometry [15–21, 23, 24]. They all used MRIs to assess quality and quantity of cartilage. The findings of each author demonstrated the existing disparities in gender anatomy. All studies agree on the better quality and greater quantity of cartilage in men than in women, except the work of Tameem et al.

Jones et al. [17] (2000) proposed the possibility that differences in cartilage formation might explain sex-related and joint compartment-related differences in the risk of osteoarthritis (OA) in the knee. They examined 92 kids randomly to measure the thickness and volume of articular cartilage. Compared to females, males had noticeably more knee cartilage. The variance in cartilage thickness and volume was statistically significant at all locations and was explained by gender in 6–36% of cases [17]. These disparities persisted even after controlling for bone density, age, BMI, physical activity, and bone area [17].

Also, Faber et al. [18] noticed gender variabilities in cartilage thickness and volumes when eighteen healthy participants, nine males and nine females, without local or systemic joint illness underwent MRIs to quantify the articular surface area, cartilage thickness, and cartilage volume. Women presented 19.9% smaller cartilage of the patella and 46.6% smaller cartilage of the medial tibia compared to men. Even though these differences were less pronounced when body weight and BMI were considered [18].

Attempting to characterize the relationship between gender, BMI, age, bone size, and cartilage degeneration rate, Ding et al. [19, 24] conducted two studies, in 2003 and 2007, that showed a significantly higher (33–42%) knee cartilage volume in men than women [19], and that the rate of cartilage deterioration in women was much greater than in males after 40 years old (medial tibia − 3.5%, lateral tibia − 2.6% and patella − 0.8% of cartilage loss per year) [24].

An average atlas of the morphology of the femoral knee cartilage was produced by Tameem et al. [15]. After adjusting for joint surface area, they found that women's cartilage was thicker (average Jacobian, a voxel-based measure of the volume that provides information on the localized volume changes) at 1.2 ± 0.078 versus 1.08 ± 0.097 [15]. The cartilage of each subject was initially normalized to the atlas for the overall size: therefore, if the cartilage scaled nonlinearly with body size, a linear global correction resulted in a bigger cartilage volume [15].

In 2014, Pachowsky et al. [23] observed that there were gender disparities in both post-matrix-associated autologous chondrocyte transplantation (MACT) zones and healthy cartilage. They also found worse quality cartilage in female with less healing capacity than male. In fact, T2 values with the MRI were significantly shorter in men than women.

Using 3 T MRI, Kumar et al. [16] quantitatively analysed the knee cartilage of three patient cohorts: young active, middle-aged without OA, and middle-aged with OA. Their findings indicated that women had higher levels of lateral articular and patellofemoral cartilage on T1ρ (or T1*rho*, used for quantitative cartilage mapping due to its sensitivity to the proteoglycans of the cartilage extracellular matrix), which indicate a lower proteoglycan content and lower tissue quality.

Moreover, Berman et al. [20] discovered gender-based morphological variations in the architecture of femoral condyles, even in patients with the same height. A stratified study of patient height and medial/lateral condylar width revealed a significant difference in condylar widths for each stratum. They also observed a statistically significant difference in articular cartilage width between males and females (31.62 ± 3.54 for males and 26.53 ± 3.70) [20].

Anthony et al. [21] examined the quantity of tibial cartilage in a group of young Australians. Men's tibial cartilage volume was found to be 13% bigger than women's [21]. Nevertheless, after adjusting for fibrinogen, fat mass, and lean body mass, the strength of this correlation declined [21].

### Hip cartilage morphometry

The work for the hip cartilage evaluation demonstrated a better quality of the cartilage of men compared to women. Nemeth et al. [22] evaluated hip cartilage by analysing thirty asymptomatic participants' right hip MRIs. The average T1ρ value was greater in women than in males (5.98 ms higher in women compared to men). Age, BMI, and the level of sports activity did not significantly affect the mean T1ρ value [22]. Additionally, women's T1ρ value standard deviations were larger than men's.

### Histological analysis

Histological quality of cartilage appears worse in women than in men. The osteoarthritic cartilage was histologically analysed only by Kaspiris et al. [14]. The study examined the frequency of cracks over the course of OA and tried to relate them to underlying cellular alterations. Three patient groups were analysed, one with knee OA, one with hip OA, and a control group of unaffected population. They categorized each group for the disease severity. Patients with moderate to severe OA exhibited a higher frequency of cartilage cracks, but those with severe OA showed them in the subchondral bone [14]. Moreover, those with a more severe histology grade had a larger frequency of cracks in the hip OA patient group. Lastly, they noticed that females often had more cartilage cracks [14].

## Discussion

Aim of the present article was to review and summarize the current literature about gender differences in hip or knee cartilage composition, and the potential implications of these differences on joint degeneration in diagnosis and therapy of osteoarthritis. Our results show that men have a significantly higher cartilage volume than women, who are more likely to develop osteoarthritis.

These results should be read in the context of their limitations. These mainly stem from the difficulty of finding manuscripts specifically focused on exploring gender-related differences, where the primary purpose and key outcome measures are explicitly stated. Most clinical research relies on standard methodologies that incorporate sex-adjusted analyses and consider gender as a confounding variable. The diverse range of studies included and their limited degree of evidence pose an additional challenge, significantly limiting the interpretability of our results.

One known risk factor for osteoarthritis (OA) is female gender: women older than 50 years of age are more likely to develop OA in the hands, hips, or knees, and it is often found to be at a more advanced stage already at clinical onset [25, 26]. The reasons behind this evidence are still unclear. Understanding the fundamental processes underlying gender-related variations and the role that gender differences play in the development of osteoarthritis (OA) would be crucial from an epidemiological, diagnostical, and treatment perspective. Indeed, identifying gender-based disparities would result in ad hoc diagnostic procedures, and customized treatment for men and women [6].

Our results showed considerable variability in the outcomes assessed by examining changes in cartilage width and structure. While no histological analysis was performed in any of the included investigations to search for variations in cartilage composition, imaging studies were used to indirectly illustrate these variations. MRI was primarily used to assess the volume, thickness, and composition of cartilage [15–21, 23, 24]; both qualitative and quantitative variations were documented. Males were often shown to have higher hip and knee cartilage volumes than females, even after adjustment for height and body weight [16, 18, 19]. According to Faber et al. [18], rather than the cartilage's thickness, the variation in cartilage volume among individuals with healthy knees is mostly caused by a difference in joint size. However, the study was unable to relate this discrepancy to the inter-individual anthropometric variabilities [18]. Another study conducted by Ding and colleagues assessed that the disparity in volume between genders can only be partially attributed to differences in body mass and bone size, and in any case, it is not impacted by the level of physical activity [19, 24].

Different studies [15, 16, 19] assessed that female’s cartilage has an higher T1ρ on MRI, which is indicative of inferior quality due to a lower proteoglycan concentration.

According to Kumar and colleagues [16], the variation in volume may be ascribed to the articular cartilage's composition as well as a distinct walking alignment mechanism. The analysis of the morphological atlas of the distal femur's articular cartilage conducted by Tameen et al. [15] revealed different load points between men and women, especially in the medial weight-bearing region and the trochlear femoral side of the patellofemoral compartment. These differences could be interpreted as a risk factor for the development of OA.

The single study on hip cartilage was carried out in 2017 by Nemeth et al. [22]. Based on MRI cartilage quantitative evaluation, they confirmed the results of other studies on knee joints [16–18, 23], founding a gender difference of 10% higher in females T1ρ values.

After the age of 50, the observed gender variations in cartilage composition become more noticeable, thus indicating a possible connection between the development of cartilage width and quality during growth and the subsequent loss of cartilage throughout adulthood [24].

In terms of cartilage development, Jones et al. [17] conducted an interesting study on healthy children. The analysis of the knees’ cartilage thickness and volume showed gender-related differences from an early age. Males had significantly more knee cartilage than females in terms of both thickness and volume [17].

Regarding cartilage loss, females have higher annual rates of change in knee cartilage volume compared to men [24]. It is hypothesized that the drastic reduction in oestrogen levels in postmenopausal women triggers and initiates OA [27, 28]. Ding et al. [24] showed that women start the cartilage loss around the age of 40, with a notable escalation after menopause, as anticipated [19]. So, many studies found that sex differences vary with age and become greater in females > 55: the reasons for this age specificity are largely unknown but may reflect the effect of oestrogen deficiency in earlier life and of menopause in later life. Our speculation is that, although oestrogen may have a modulating effect on cartilage, the effects of sex hormones and growth factors in mediating such and age-sex interaction in OA risk are still poorly understood and need further investigation.

Furthermore, women undergoing hormone replacement therapy demonstrated a diminished rate of cartilage degeneration [29]. This raises the possibility of preventative measures for women in the onset of OA. However, hormonal fluctuations are not the sole cause of cartilage loss, and the reason behind this cartilage disparity between females and males remains unknown and needs to be studied.

In the authors’ opinion, this article shows how deeply gender differences may affect both medicine and surgery; some authors, considering the morphological and biomechanical differences between men and women, utilize gender-specific customized prosthetic implants with very good outcome [30, 31]. This suggests how medicine is moving towards an increasingly deeper understanding of the biological mechanisms underlying pathologies, aiming to prevent them where possible, or, when prevention is not possible, to treat them in the most personalized manner.

## Conclusions

Men exhibit significantly higher knee and hip cartilage volumes than women. Thus, women are significantly more likely than men to develop osteoarthritis, especially with ageing. These differences seem to be only partially influenced by body and bone size, suggesting the need for further research in this area. Moreover, these gender disparities become more pronounced after the age of fifty, indicating that gender-related variations in cartilage volume throughout adulthood are shaped by both cartilage loss and development during childhood and adolescence. Currently, in the age of precision medicine, there are no gender-specific procedures available to address and compare the progression of end-stage osteoarthritis.

## Declarations

### Protocol registration

PROSPERO does not accept scoping reviews, literature reviews or mapping reviews. For these reasons our review and its protocol are not registered.
